# Summary: EANM/SNMMI Release Joint Guideline/Procedure Standard for [^18^F]FDG Hybrid PET Use in Infection and Inflammation in Adults, Version 2.0

**DOI:** 10.2967/jnumed.125.269538

**Published:** 2025-03

**Authors:** Olivier Gheysens, Matthieu Pelletier-Galarneau, Giorgio Treglia, Ora Israel, Francois Jamar, Gad Abikhzer

**Affiliations:** 1 Department of Nuclear Medicine, Cliniques Universitaires Saint-Luc and Institute of Clinical and Experimental Research, Université Catholique de Louvain, Brussels, Belgium;; 2 Department of Medical Imaging, Montreal Heart Institute, Montreal, Quebec, Canada;; 3 Nuclear Medicine, Imaging Institute of Southern Switzerland, Ente Ospedaliero Cantonale, Bellinzona, Switzerland; Department of Nuclear Medicine and Molecular Imaging, Lausanne University Hospital, University of Lausanne, Lausanne, Switzerland; and Faculty of Biomedical Sciences, Università della Svizzera Italiana, Lugano, Switzerland;; 4 Rappaport School of Medicine, Technion-Israel Institute of Technology, Haifa, Israel; and; 5 Department of Radiology and Nuclear Medicine, Faculty of Medicine and Health Sciences, Jewish General Hospital, McGill University, Montreal, Quebec, Canada

A joint task force of subject-matter experts from the European Association of Nuclear Medicine (EANM) and the Society of Nuclear Medicine and Molecular Imaging (SNMMI) recently released a revised and updated guideline/procedure standard for indications and protocols for hybrid [^18^F]FDG imaging in infection and inflammation in the adult population. The complete document was posted to the SNMMI website on June 7 and published on October 10 ahead of print in the *European Journal of Nuclear Medicine and Molecular Imaging* (1,2). Use of [^18^F]FDG imaging in these indications has grown rapidly since the first version of the guideline was published in 2013 (3), as has the body of evidence-based literature. Because hybrid [^18^F]FDG metabolic imaging is today generally the method of choice for most infection and inflammation indications, updating these guidelines with relevant literature over the intervening decade was deemed timely. The authors’ search included all pertinent systematic reviews and metaanalyses, focusing on PET/CT rather than standalone PET. When systematic reviews and metaanalyses were not available for specific indications, results from prospective or retrospective studies were considered. The publication complements many related EANM and SNMMI guidelines/procedure standards, as cited in the extensive supporting references, but where possible avoids duplication of more detailed recommendations on specific topics (such as quality control, general acquisition parameters, radiopharmaceutical characteristics, and general basic and clinical aspects of [^18^F]FDG imaging) addressed elsewhere.

For each of 19 indications defined and covered in the document, the authors provide specific protocol points, interpretation criteria, diagnostic performance points (with extended tabular material and references), and areas for potential research. Fever/inflammation of unknown origin is the first topic covered. Indications in infection include bacteremia/septicemia and evaluation of metastatic infection/septic embolism, suspected spondylodiskitis with and without spinal hardware, suspected osteomyelitis in noncomplicated bone and septic arthritis, suspected osteomyelitis in complicated bone, diabetic foot infections, periprosthetic joint infections, prosthetic valve endocarditis, native valve endocarditis, cardiac implantable electronic device infection, ventricular assist device infection, vascular graft and endograft infections, suspected infected liver and kidney cysts, invasive fungal infections, and tuberculosis and other mycobacterioses. Indications in inflammation include large-vessel vasculitis and polymyalgia rheumatica, sarcoidosis, inflammatory bowel diseases, and retroperitoneal fibrosis and IgG4-related disease. Sufficient evidence was not found to support inclusion of coronavirus disease 2019 (including long coronavirus disease), interstitial lung diseases, or inflammatory arthropathies and myopathies among the indications published in this guideline/procedure standard, although the authors acknowledge the possibility that such supporting evidence may be available in the future.

The last section of the document summarizes recommendations relevant to [^18^F]FDG imaging of infection and inflammation across all indications: procedures for requests for the examination, preparation and precautions for patients with a range of special conditions and medications, radiopharmaceutical administration, radiation exposure, image acquisition protocol, and image analysis and interpretation.

The updated guideline/procedure standard concludes with a thoughtful analysis of gaps in the current literature and suggested research topics to address these gaps and contribute to the enhancement of future clinical practice. Among these recommended topics are assessing the potential role for late imaging (90–180 min after [^18^F]FDG injection) in selected indications (e.g., osteomyelitis and cardiovascular imaging) to improve image quality through higher target-to-background ratios; assessing the role of electrocardiogram-gated acquisition in suspected endocarditis; assessing the added value and potential improvement of diagnostic yield using intravenous iodinated contrast medium in selected indications; defining the role and thresholds for SUV or other quantitative or semiquantitative indices to differentiate infection from sterile inflammation or malignant processes; determining whether diagnostic accuracy is improved with new digital or large-field-of-view hybrid PET systems, particularly in the evaluation of small lesions, while also reducing administered radiotracer activity; exploring the potential added value of PET/MRI for assessment of infectious processes in general and specifically for indications such as spondylodiskitis, diabetic foot infection, osteomyelitis, polycystic disease, cardiac sarcoidosis, cranial artery vasculitis, and inflammatory bowel disease; comparing the diagnostic accuracy and feasibility of [^18^F]FDG imaging with that of other modalities (e.g., white blood cell SPECT/CT and MRI) in various indications; understanding the impact on diagnostic accuracy of antibiotic therapy and its duration before imaging; assessing the potential applications of [^18^F]FDG PET in monitoring therapy response; identifying optimal time points for integrating [^18^F]FDG PET/CT in the diagnostic workup of infectious and inflammatory processes in terms of cost-effectiveness; and evaluating the potential role of artificial intelligence for [^18^F]FDG PET/CT in assessment of infectious and inflammatory diseases.

## DISCLOSURE

No potential conflict of interest relevant to this article was reported.
